# Testosterone therapy for women with low sexual desire: a position statement from the Brazilian Society of Endocrinology and Metabolism

**DOI:** 10.20945/2359-3997000000152

**Published:** 2019-07-12

**Authors:** Rita V. Weiss, Alexandre Hohl, Amanda Athayde, Dolores Pardini, Larissa Gomes, Monica de Oliveira, Ricardo Meirelles, Ruth Clapauch, Poli Mara Spritzer

**Affiliations:** 1 Pontifícia Universidade Católica do Rio de Janeiro Instituto Estadual de Diabetes e Endocrinologia Luiz Capriglione Pontifícia Universidade Católica do Rio de Janeiro Escola Médica de Pós-Graduação em Endocrinologia Rio de Janeiro RJ Brasil Instituto Estadual de Diabetes e Endocrinologia Luiz Capriglione, Pontifícia Universidade Católica do Rio de Janeiro, Escola Médica de Pós-Graduação em Endocrinologia (IEDE-PUC/RJ), Rio de Janeiro, RJ, Brasil; 2 Universidade Federal de Santa Catarina Departamento de Medicina Interna Hospital Universitário Universidade Federal de Santa Catarina Florianópolis SC Brasil Serviço de Endocrinologia e Metabolismo, Departamento de Medicina Interna, Hospital Universitário (HU), Universidade Federal de Santa Catarina (UFSC), Florianópolis, SC, Brasil; 3 Universidade Federal do Rio de Janeiro Universidade Federal do Rio de Janeiro Rio de Janeiro RJ Brasil Universidade Federal do Rio de Janeiro (UFRJ), Rio de Janeiro, RJ, Brasil; 4 Universidade Federal de São Paulo Universidade Federal de São Paulo São Paulo SP Brasil Disciplina de Endocrinologia, Universidade Federal de São Paulo (UNIFESP), São Paulo, SP, Brasil; 5 Universidade de São Paulo Faculdade de Medicina Hospital de Clínicas Universidade de São Paulo São Paulo SP Brasil Disciplina de Endocrinologia, Faculdade de Medicina, Hospital de Clínicas, Universidade de São Paulo (USP), São Paulo, SP, Brasil; 6 Instituto de Medicina Integral Professor Fernando Figueira Instituto de Medicina Integral Professor Fernando Figueira Recife PE Brasil Instituto de Medicina Integral Professor Fernando Figueira, Recife, PE, Brasil; 7 Departamento de Fisiologia Endócrina e Fisiologia Laboratório de Pesquisas Clínicas e Experimentais em Biologia Vascular Rio de Janeiro RJ Brasil Departamento de Fisiologia Endócrina e Fisiologia e Laboratório de Pesquisas Clínicas e Experimentais em Biologia Vascular (BIOVASC), Rio de Janeiro, RJ, Brasil; 8 Universidade Federal do Rio Grande do Sul Hospital de Clínicas de Porto Alegre Departamento de Fisiologia Universidade Federal do Rio Grande do Sul Porto Alegre RS Brasil Unidade de Endocrinologia Ginecológica, Serviço de Endocrinologia, Hospital de Clínicas de Porto Alegre e Departamento de Fisiologia, Universidade Federal do Rio Grande do Sul (UFRGS), Porto Alegre, RS, Brasil

**Keywords:** Testosterone therapy, female sexual disorder, position

## Abstract

**Objective:**

To summarize current evidence regarding testosterone treatment for women with low sexual desire.

**Materials and methods:**

The Female Endocrinology and Andrology Department of the Brazilian Society of Endocrinology and Metabolism invited nine experts to review the physiology of testosterone secretion and the use, misuse, and side effects of exogenous testosterone therapy in women, based on the available literature and guidelines and statements from international societies.

**Results:**

Low sexual desire is a common complaint in clinical practice, especially in postmenopausal women, and may negatively interfere with quality of life. Testosterone seems to exert a positive effect on sexual desire in women with sexual dysfunction, despite a small magnitude of effect, a lack of long-term safety data, and insufficient evidence to make a broad recommendation for testosterone therapy. Furthermore, there are currently no testosterone formulations approved for women by the relevant regulatory agencies in the United States, Brazil, and most other countries, and testosterone formulations approved for men are not recommended for use by women.

**Conclusion:**

Therefore, testosterone therapy might be considered if other strategies fail, but the risks and benefits must be discussed with the patient before prescription. Arch Endocrinol Metab. 2019;63(3):190-8

## INTRODUCTION

Sexual function in women is a complex issue, impacted upon by several factors. Female testosterone levels decrease slightly during the reproductive years, but do not change substantially after menopause, because the ovaries continue to produce androgens after depletion of follicular capital (1,2). Reduced androgen levels have been frequently associated with reduced libido in women, mainly in the peri- and postmenopausal periods (1-4). While testosterone has been frequently prescribed to women with low sexual desire, there is no evidence of a consistent association between low testosterone levels and low libido, nor of consistent improvement in sexual problems when testosterone therapy is administered. Therefore, testosterone therapy is not routinely recommended for women with low androgen levels caused by adrenal insufficiency, hypopituitarism, or surgical menopause, nor to enhance cardio-metabolic parameters, cognitive health, or general well-being in healthy women. In fact, the use of androgens in postmenopausal women remains controversial, with strong positions being taken by a variety of sexual medicine experts and professional societies (5-9).

In 1997 and 1998, two meetings were held in Cape Cod, Massachusetts, to discuss the clinical detection and management of female sexual dysfunction. These two meetings, and their subsequent iterations, would lead to the formation of the International Society for the Study of Women’s Sexual Health (ISSWSH) in 2001 (10). Later in 2002, the Princeton Consensus proposed the existence of a female androgen insufficiency syndrome, defined as a pattern of clinical symptoms in the presence of decreased bioavailable testosterone and normal estrogen status (11). The diagnostic criteria were criticized because the syndrome definition implied that sexual dysfunction, the predominant clinical finding, was a consequence of androgen insufficiency (12). In 2005, a study revealed that, while serum androgen levels decline steeply in the early reproductive years, they do not vary as a direct consequence of natural menopause. The investigators pointed out that the significant age-related variations in androgens must be taken into account when normal ranges are reported and in studies on the role of androgens in women (1).

Recently, a systematic review assessed the available evidence regarding the evaluation of serum testosterone levels and testosterone treatment for premenopausal women with low libido from 1995 to 2015. Nine out of ten studies failed to find a correlation between total testosterone levels and sexual desire, and four small studies showed little, if any, improvement in libido when compared to placebo (13). Therefore, the present position statement was developed to review the existing literature on the off-label use of testosterone to treat low sexual desire in women.

## MATERIALS AND METHODS

Nine members of the Female Endocrinology and Andrology Department of the Brazilian Society of Endocrinology and Metabolism, experts in clinical practice, clinical research and/or translational research in the field, were asked to critically review the topic of testosterone use and develop a position statement on testosterone therapy for women with low sexual desire. MEDLINE and SciELO/LILACS databases were searched for relevant studies and clinical practice guidelines regarding testosterone therapy in women. The level of evidence of each publication was graded using the Oxford Centre for Evidence-Based Medicine recommendations (14). In the present position statement, these levels of evidence are reported as: A: experimental or observational studies with consistent results; B: experimental or observational studies with less consistent results; C: case reports (uncontrolled studies); D: opinion lacking critical evaluation or based on guidelines, physiological studies, or animal models (15).

### Testosterone levels in women across the lifespan

During childhood, testosterone levels are very low. At puberty, kisspeptin triggers pulsatile gonadotropin releasing hormone (GnRH) secretion in the medial basal hypothalamus and preoptic area (16), enhancing luteinizing hormone (LH) secretion and consequently stimulating estradiol and testosterone secretion. Upon puberty and during the reproductive years, mainly in young adult women, testosterone is produced equally by the ovarian theca cells and the adrenal cortex, accounting for approximately 300 mcg daily (17,18).

Only one-third of circulating testosterone results from direct ovarian and adrenal secretion; the remaining two-thirds arise from peripheral conversion of precursors, including delta 4-androstenedione (A4) and DHEA, in non-steroid-producing tissues. Testosterone is converted to estradiol by aromatase and to dihydrotestosterone (DHT) by 5-alpha reductase, in target tissues as well as in the periphery (mainly in adipose tissue) (19).

As in men, testosterone production in women follows a circadian rhythm, with higher levels in the morning and a nadir around midnight (20). Moreover, a modest mid-cycle testosterone peak, coincident with the LH surge in ovulatory cycles, has been confirmed by recent high-sensitivity assays (21). Apart from this mid-cycle peak, testosterone levels in the follicular and luteal phases seem to be stable (1,21,22). During the late reproductive years, the increment of anovulatory cycles leads to a failure of this mid-cycle rise, despite preservation of baseline physiological ovarian testosterone secretion at other phases of the cycle (3). Aging promotes a progressive reduction in testosterone production by the ovaries and adrenal glands (Table 1). At that stage, the peripheral conversion of A4 and DHEA into testosterone becomes more important (23-25). DHEA serves as an intermediate steroid within the testosterone and estradiol biosynthetic pathways. Peripheral tissues involved with DHEA/DHEA-S uptake from the circulation typically generate more potent androgens and estrogens that act locally (26,27).

**Table 1:** Testosterone levels across the lifespan in a reference group of Australian women

Age (years)	18-24	25-34	35-44	45-54	55-64	65-75
Total testosterone (25-83 ng/dL) (direct manual RIA method)	45.53	31.98	26.9	23.34	19.0	20.46
Free testosterone, calculated (370-1,110 pg/dL) Sodergard equation (based on total testosterone, SHBG, and albumin levels)	680.4	497.1	393.9	340.6	311.5	281.2

Modified from Davison and cols. (1).

During the menopause transition, the decline in circulating estrogen is more pronounced than the decline in androgen levels, and the ensuing gradual increase in the testosterone to estradiol ratio (T/E) leads to a relative hyperandrogenic state (28). This relative androgen excess status has been associated with central abdominal adiposity and the metabolic syndrome (28-30). Also, serum levels of sex-hormone binding protein (SHBG) contribute to testosterone transport and availability, limiting the amount of free and bioactive testosterone (1). Thus, the relative hyperandrogenic status of menopause is associated with lower SHBG levels, resulting in increased free testosterone levels (24,25).

Surgical menopause induced by bilateral oophorectomy, however, is associated with a different pattern. In an epidemiological cross-sectional survey, Australian women aged 55 years or older who had undergone bilateral oophorectomy showed significantly lower total and free testosterone levels than age-matched controls with both ovaries. The same study observed a very gradual decrease in the ovarian and adrenal function of 1,423 Australian women starting in the early reproductive years: from 18 to 75 years of age, total testosterone decreased 55%, free testosterone decreased 49%, and DHEA-S decreased 77% (1).

### Testosterone therapy for sexual desire dysfunction

The diagnosis of sexual desire dysfunction has historically been guided by the criteria set out in the Diagnostic and Statistical Manual of Mental Disorders (DSM). From 1980 to 2013, the diagnosis of hypoactive sexual desire disorder (HSDD) depended on the symptom of absent or reduced desire for sexual activity and lack of sexual fantasies, with associated clinically significant distress. Recently, the DSM-5 introduced broader criteria for the diagnosis of sexual desire dysfunction, and merged desire and arousal into a single category, assigning this condition the diagnostic entity “female sexual interest/arousal disorder” (SIAD) (31). A recent study has reported that around 73% of women with a diagnosis of HSDD also met criteria for SIAD (32). However, it is important to point out that the DSM-5 is a psychiatric compendium, and is not intended for clinical use by the disciplines that care for sexual medicine patients in clinical settings. In this sense, both the International Consultation on Sexual Medicine publications and the International Society for the Study of Women’s Sexual Health (ISSWSH) Nomenclature indicate that current evidence does not support the merger of desire and arousal into a single category, supporting the maintenance of an HSDD diagnostic category (33-35). A recent publication (36) indicates that ICD-11 intends to preserve HSDD as a separate category for coding (Table 2).

**Table 2:** Proposed classification of female sexual dysfunction in ICD-11 and current ICD-10, and DSM-5 definitions

Proposed ICD-11	ICD-10	DSM-5
Hypoactive sexual desire and dysfunction	Absence or loss of sexual desire	Female sexual interest/arousal disorder
Category: Female sexual arousal dysfunction obs: the psychological component of arousal that corresponds to the ICD-10 *Lack of sexual enjoyment* is also included in this proposed classification	Failure of genital response; lack of sexual enjoyment	Female sexual interest/arousal disorder

Adapted from Reed et al. (36).

A diagnosis of HSDD must include any of the following symptoms, with a duration of at least 6 months: “1) lack of motivation for sexual activity, either by decreased or absent spontaneous desire (sexual thoughts or fantasies) or by decreased or absent responsive desire to erotic cues and stimulation or inability to maintain desire or interest through sexual activity; 2) loss of desire to initiate or participate in sexual activity, including behavioral responses such as avoidance of situations that could lead to sexual activity, that is not secondary to sexual pain disorders” (37, p. 468). These manifestations are “combined with clinically significant personal distress that includes frustration, grief, guilt, incompetence, loss, sadness, sorrow, or worry” (37, p. 468). A sexual history and/or the use of a validated instrument, such as the Decreased Sexual Desire Screener (DSDS) (38), are recommended for confirming the diagnosis and determining the type of HSDD (33,37). In women going through the natural menopausal transition, a comprehensive, longitudinal study has found that prior function and relationship factors have a stronger impact on female sexual function than hormone concentrations (A) (39).

Clinical management of HSDD encompasses sex therapy, central nervous system drugs, and hormonal agents, and should always consider menopausal status. Concerning hormone therapy, testosterone is currently only approved for that purpose in Australia; it remains an off-label treatment in other countries. Transdermal testosterone has been widely studied to treat HSDD. A systematic review of seven randomized trials, enrolling more than 3,000 women, evaluated the efficacy of a 300 mcg testosterone patch and found that the testosterone group had more satisfying sexual episodes, sexual activity, orgasm, desire, and improvement in Personal Distress Scale scores, but also increased acne and hair growth, suggesting a supraphysiologic dose (40). According to the authors, short-term transdermal testosterone was efficient in improving sexual function in naturally and surgically menopausal women affected by HSDD, both on or off estrogen-progestin hormone therapy (38). This evidence has been incorporated into the clinical practice guidelines issued by at least four Societies (A) (3,37,41,42).

Therefore, in the presence of a proper HSDD diagnosis, once informed consent is obtained from the patient, an individualized trial of testosterone therapy for 3 to 6 months could be suggested, aiming at mid-normal premenopausal testosterone levels during treatment**.** The existing guidelines state that in the absence of clear improvement and if adverse events are observed at 6 months, the use of testosterone should be ceased (D) (3,37,41). There are no safety and efficacy data for testosterone therapy in women with HSDD beyond 24 months (3,37,41,42), although one single 4-year open-label extension trial in surgically menopausal women with HSDD showed no relevant safety issues during this period (43). While non-oral preparations (such as a transdermal patch, gel, or cream) are recommended for therapy in women, such preparations are not available in most countries, including Brazil. It is important to note that testosterone preparations formulated for men are not recommended for use by women, due to lack of data concerning efficacy and safety of these preparations in women (3,37,42).

In women who are receiving a trial of testosterone therapy, testosterone levels should be measured at baseline and 3-6 weeks after starting treatment, especially to avoid supraphysiologic concentrations, since response to therapy does not correlate with testosterone levels (B) (3,34,37). During ongoing testosterone therapy, signs of clinical androgen excess and laboratory testosterone levels should be assessed every 6 months to monitor potential side effects.

Current evidence from women with adrenal insufficiency (44) or normal adrenal function (45) does not support DHEA use for HSDD treatment (A). Besides, two drug combinations, based on known neurobiological mechanisms that are critically important in sexual excitation and inhibition (46,47), are in development for low sexual desire in women. One consists of a tablet that combines a testosterone coating for sublingual administration and an inner-core component containing the phosphodiesterase type 5 inhibitor sildenafil. Sublingual testosterone could potentially increase the brain’s responses to sexual cues, leading to increases in sexual motivation, while sildenafil could increase genital vasocongestion (46,47). The other drug is a tablet which combines an inner-core component containing the 5-hydroxytryptamine (5-HT)1A receptor agonist buspirone coated with a delayed-release matrix impregnated with testosterone, which could ensure that the pharmacologic effects of both components coincide (46,47). However, these investigational drugs have not advanced yet to Phase III clinical trials and further progress are needed before considering as a treatment option.

### Laboratory evaluation of testosterone levels

Reliable testosterone assays are essential in the diagnosis and management of a number of clinical conditions in females, including precocious pubarche, androgen-secreting tumors, and the polycystic ovary syndrome (PCOS) (48). However, most testosterone assays have poor sensitivity and accuracy in the female ranges (normal range, 20-60 ng/dL). Circulating testosterone levels in women vary according to reproductive status, phase of the menstrual cycle, and time of day (49). There are no universally normal ranges applicable to all ages and sexes, and, in fact, there is no testosterone standard on which to base an assay. Only 1-3% of testosterone is not bound to plasma proteins, raising questions about which measure of testosterone is the most clinically useful – total (TT) or free testosterone (FT) (50).

The commonly used methods for measuring TT or FT are immunoassays and mass spectrometry (MS). Automated chemiluminescence is the most common method overall, although it has lower accuracy. In immunoassays, extraction of testosterone and purification by chromatography before the assay decreases cross-reactivity with other steroids and improves the accuracy of the method (49); however, these procedures are unwieldy for commercial use (48). Despite significant advances to improve the accuracy and precision of currently available assays, limited comparability exists between assays at the lower and upper extremes of the testosterone range. Because of this lack of comparability, there is no current standard immunoassay for the assessment of total testosterone levels (49).

In turn, extraction and high-performance chromatography followed by MS is currently considered the gold standard for measuring total testosterone (3,49,51,52). However, obtaining and maintaining a mass spectrometer is expensive, and sample throughput is generally slower and more costly than with automated immunoassays. Moreover, standardization is still needed to ensure good comparability of results across laboratories.

While TT measurement may not be ideal in the context of evaluating androgen production and availability, FT determination also poses a concern due to its low accuracy for values in the female range. Quantitation of FT levels by dialysis is perhaps the most accurate test for measuring androgens in women, but also the most costly, and is not performed by most laboratories. Ideally, testosterone measurements should be obtained in the morning hours and in the follicular phase of the menstrual cycle in normally cycling premenopausal women.

### Testosterone therapy: side effects and long-term safety

The main side effects of testosterone therapy in women are related to masculinizing symptoms and abnormal endometrial bleeding, but breast cancer and cardiovascular risk are also a reason for concern (37,53). The effects are dose-dependent; supraphysiologic doses must always be avoided.

The most common clinical side effects are acne, hirsutism, androgenic alopecia, and deepening of the voice. Clitoromegaly has not emerged as a frequent side effect at physiological doses (41,42,53). In the APHRODITE trial, a randomized study that used testosterone therapy at doses ranging from 150-300 µg/d, dose was not associated with increased rates of acne, voice change or alopecia over 12 months. However, a higher rate of terminal hair growth was observed at the 300 µg/d dose (54).

Androgen excess is usually associated with endometrial atrophy when given without concomitant estrogen. In the APHRODITE trial, women with endometrial bleeding who were exposed to the high study testosterone dose (300 µg/d) were found to have endometrial atrophy on endometrium biopsy (55). However, as most androgens can be aromatized to estrogens by aromatase, endometrial abnormalities associated with high estrogen concentrations, such as endometrial hyperplasia, dysplasia, and cancer, could also be expected during testosterone therapy (49,55).

The effects of testosterone therapy on the breast are conflicting (3,56,57). Testosterone seems to decrease breast tissue proliferation and prevent the pro-apoptotic effect mediated by estrogen (56). Patients on estrogen therapy did not show mammographic density changes, and no breast cell proliferation was observed when testosterone was added (58). Data from the Women’s Health Initiative (WHI), an observational study in which a combination of estrogen and testosterone therapy was used, show a non-significant impact on invasive breast cancer risk (adjusted HR 1.42; 95%CI, 0.95-2.11) (59).

Regarding cardiovascular and metabolic outcomes, the relationship between endogenous postmenopausal hormone levels and atherosclerosis is unclear (60-62). Oral testosterone therapy seems to negatively impact lipid profile, usually decreasing HDL and increasing LDL. However, parenteral and transdermal androgens do not seem to have this effect (60). A systematic review did not find adverse effects of testosterone therapy on blood pressure, vascular reactivity, blood viscosity, insulin sensitivity, hemoglobin concentration, or coagulation factors in postmenopausal women (63). One study showed an inverse correlation between circulating androgen levels and arterial intima-media thickness assessed by carotid ultrasonography in postmenopausal women. This result provides preliminary evidence suggesting a protective effect of androgens against the development of atherosclerosis (64). However, these data were obtained from women with a lower risk of carotid artery atherosclerosis and considering physiologic testosterone levels, and cannot be extrapolated to exogenous testosterone therapy (65).

Therefore, the role of testosterone in breast cancer and cardiovascular disease pathophysiology requires further elucidation, and evidence of safety from long-term studies is lacking.

### Testosterone misuse and abuse

The first studies to investigate the ability of supraphysiologic doses of testosterone to increase libido in postmenopausal women were conducted by Salmon and Geist, in 1943 (66). These studies showed increased libido with testosterone therapy in this population, leading to an increased interest in hormonal treatment with testosterone for lower libido in women.

Testosterone therapy could potentially be prescribed to women with HSDD, but no testosterone formulation has been approved for females by the regulatory agencies of the United States, Brazil, or most other countries. Consequently, testosterone formulations approved for men have been (mis)used off-label.

Testosterone derivatives have distinct levels of relative anabolic and androgenic activities, exerting their ergogenic and cosmetic effects (67). Exogenous androgens can increase muscle mass and strength in athletes, regardless of gender or age. Therefore, since the early 1970s, the International Olympic Committee has banned the use of anabolic-androgenic steroids (AAS) by male and female athletes (68). However, androgens continue to be used abusively as ergogenic drugs in sports. In 2006, 34 international laboratories accredited by the International Anti-Doping Agency recorded 4,332 positive findings in tests, of which 45% were due to use of androgens (67).

Furthermore, the aesthetic misuse of testosterone in non-athletes has been growing dramatically and uncontrolledly, and a clear increase in the use of these steroids by women has been observed. However, in the current medical literature, the vast majority of research data on the use of anabolic steroids in women comes from athletes (69).

Randomized studies exploring the effects of AAS in women are scarce; case reports remain the leading source of information in this regard. The type and intensity of adverse effects depend on the AAS used, its dose, and the duration of use. Also, oral formulations can be hepatotoxic. AAS administration in women can induce masculinization, with development of acne vulgaris, change in libido, and deepening voice as the adverse effects most often seen in the first weeks of anabolic steroid use. Prolonged AAS administration can induce hair loss (alopecia), changes in pubic hair growth, smaller breasts, and clitoromegaly (A). AAS use can suppress pituitary LH and FSH secretion, leading to menstrual irregularities and even infertility. Also, in some studies, female athletes reported increased aggressiveness with the use of steroids (70). Dyslipidemia, hypertension, arrhythmia, coagulation disorders, fibrosis, and cardiac hypertrophy have been observed (71,72).

The abuse of androgens in sports and in the community, for aesthetic purposes, remains a major concern. AAS abuse in the general population requires well-designed psychological prevention programs aiming to deter teenagers from future abuse of androgens. However, to date, such programs have proven effective only to increase knowledge, but not to reduce the intention to abuse or abusive behavior. In general, prevention of AAS abuse requires both demand-side and supply-side reductions, involving not only specific education for women of all ages, with attention to psychosocial predisposing factors, but also enforcement of laws to curb illegal networks that enable and maintain AAS abuse.

In conclusion, female sexual dysfunction is a common complaint in clinical practice, especially in postmenopausal women, and may have a negative impact on quality of life. Testosterone seems to exert a positive effect on sexual desire in women with HSDD, despite a small magnitude of effect, a lack of long-term safety data, and insufficient evidence for a broad recommendation of testosterone therapy, as also acknowledged in previous clinical guidelines (3,37,42). In addition, there are currently no testosterone formulations approved for women by regulatory agencies in the United States, Brazil, and most countries. Finally, testosterone formulations approved for men are not recommended for use by women. Therefore, when considering testosterone therapy, all risks and benefits should be thoroughly discussed with the patient before prescription.
